# Supplementary figures and images for: Public health policies for children and youth with special health care needs in OECD member countries and Brazil: A scoping review protocol
Source: PLoS One. 2023 Oct 11;18(10):e0287939. doi: 10.1371/journal.pone.0287939 (PMC10566681; doi:10.1371/journal.pone.0287939)

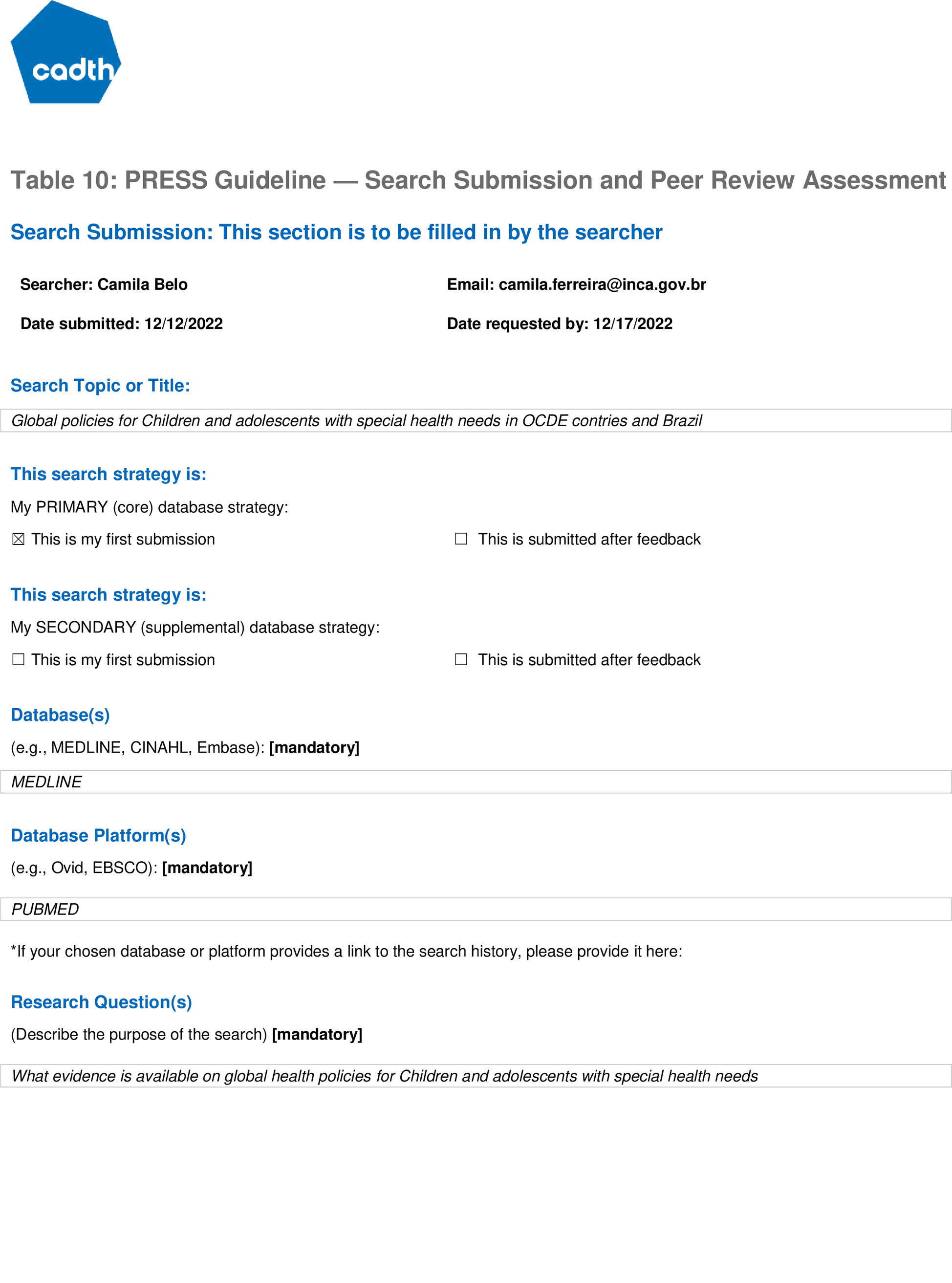

Supplement: S1 Fig — (TIF) [file pone.0287939.s002.tif]

**S2 Fig. Peer Review of Electronic Search Strategies (PRESS) guideline checklist.**
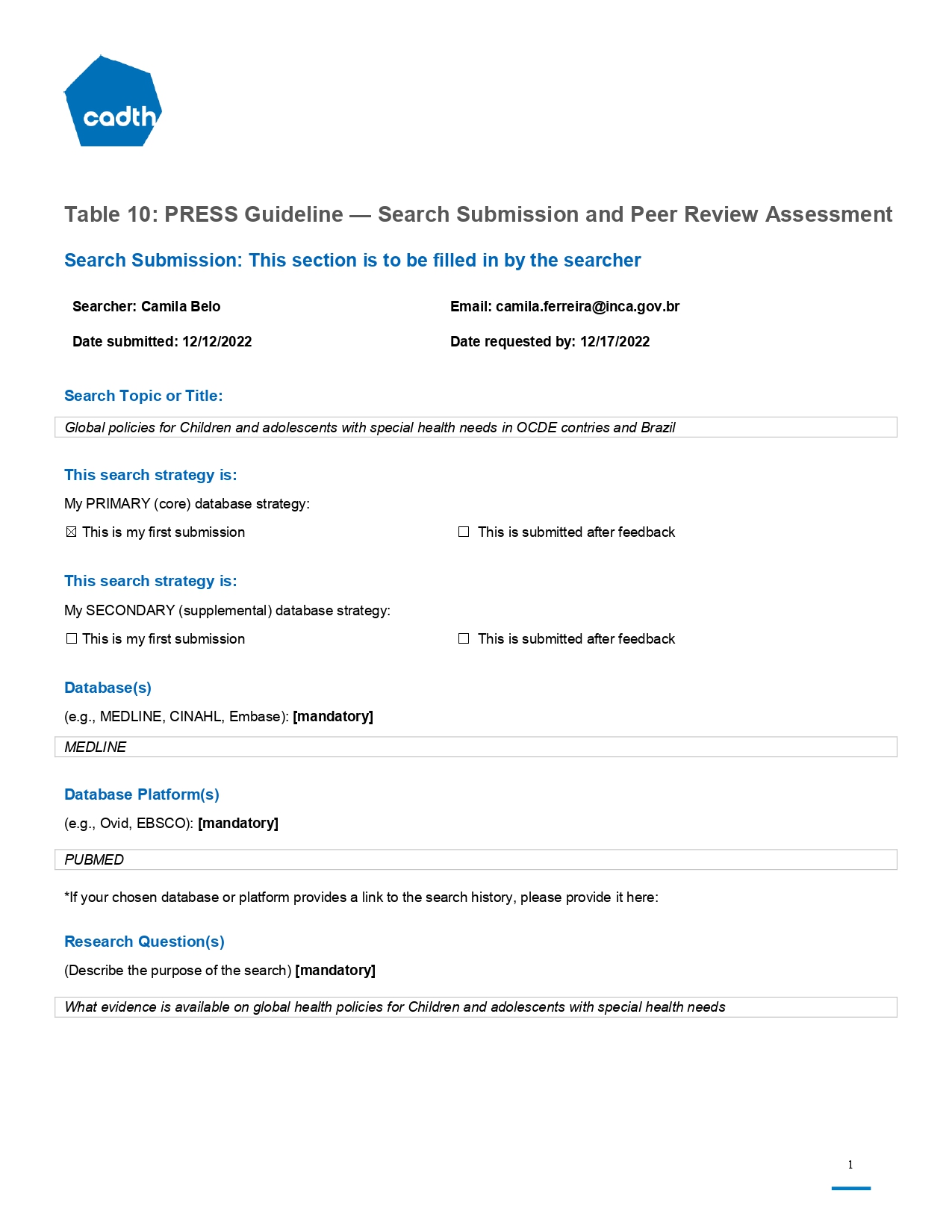

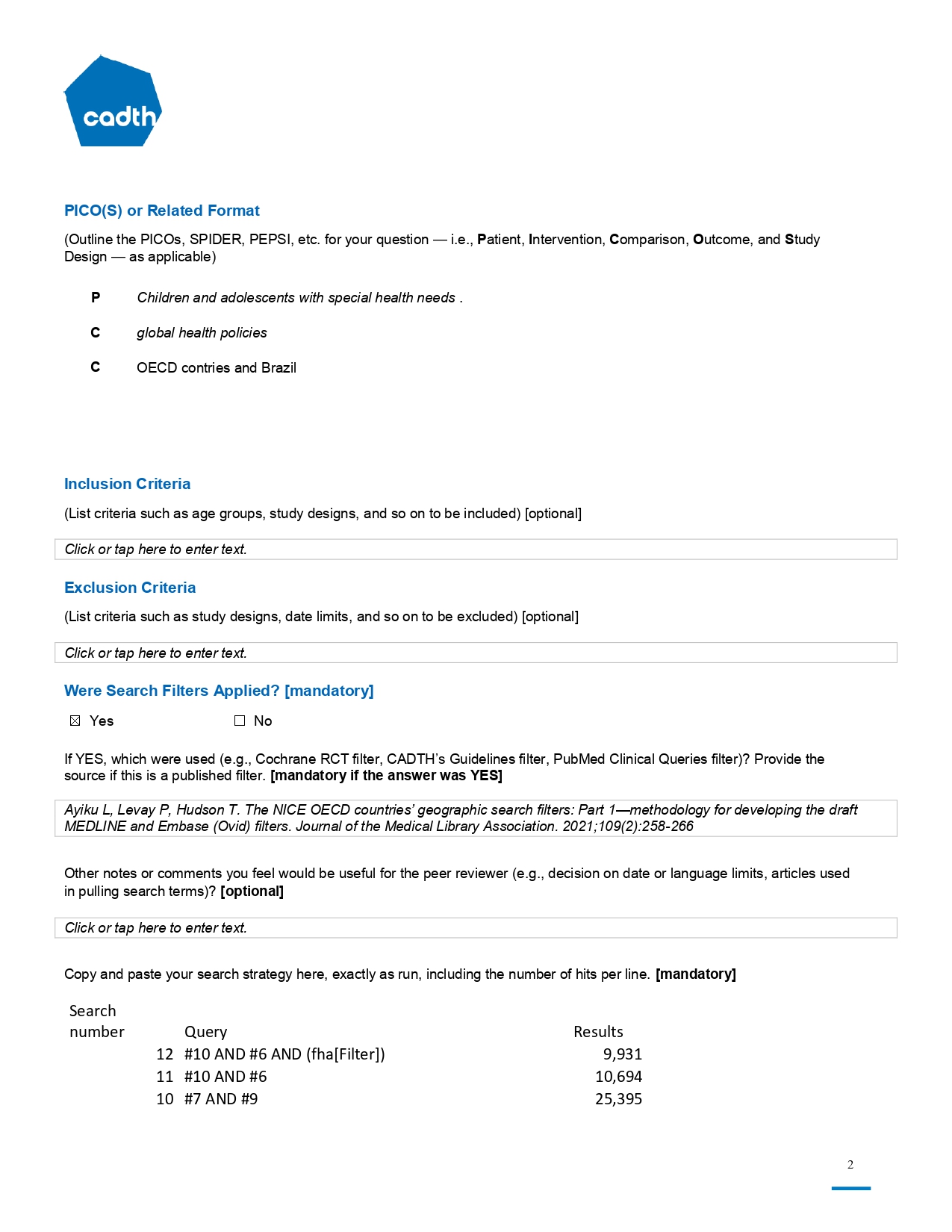

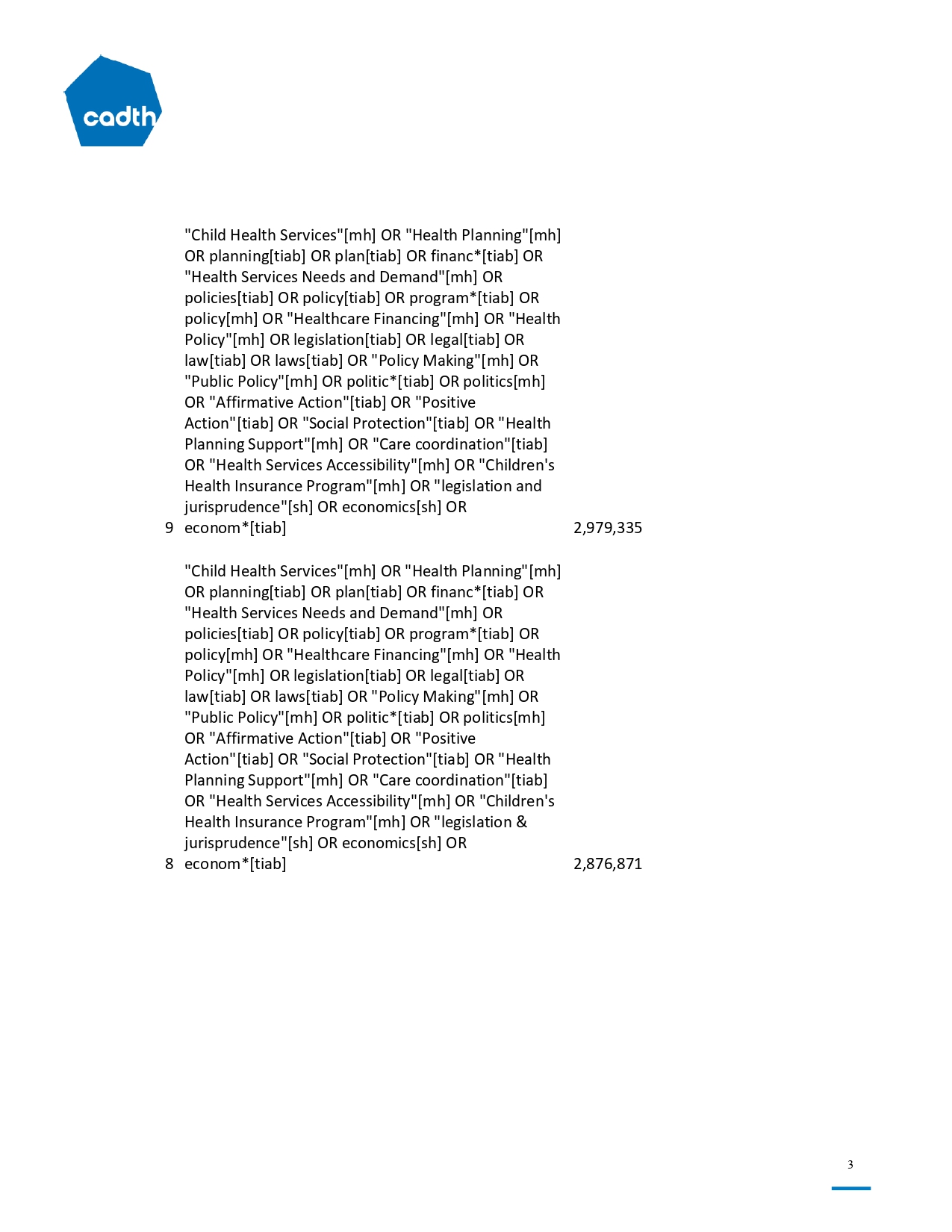

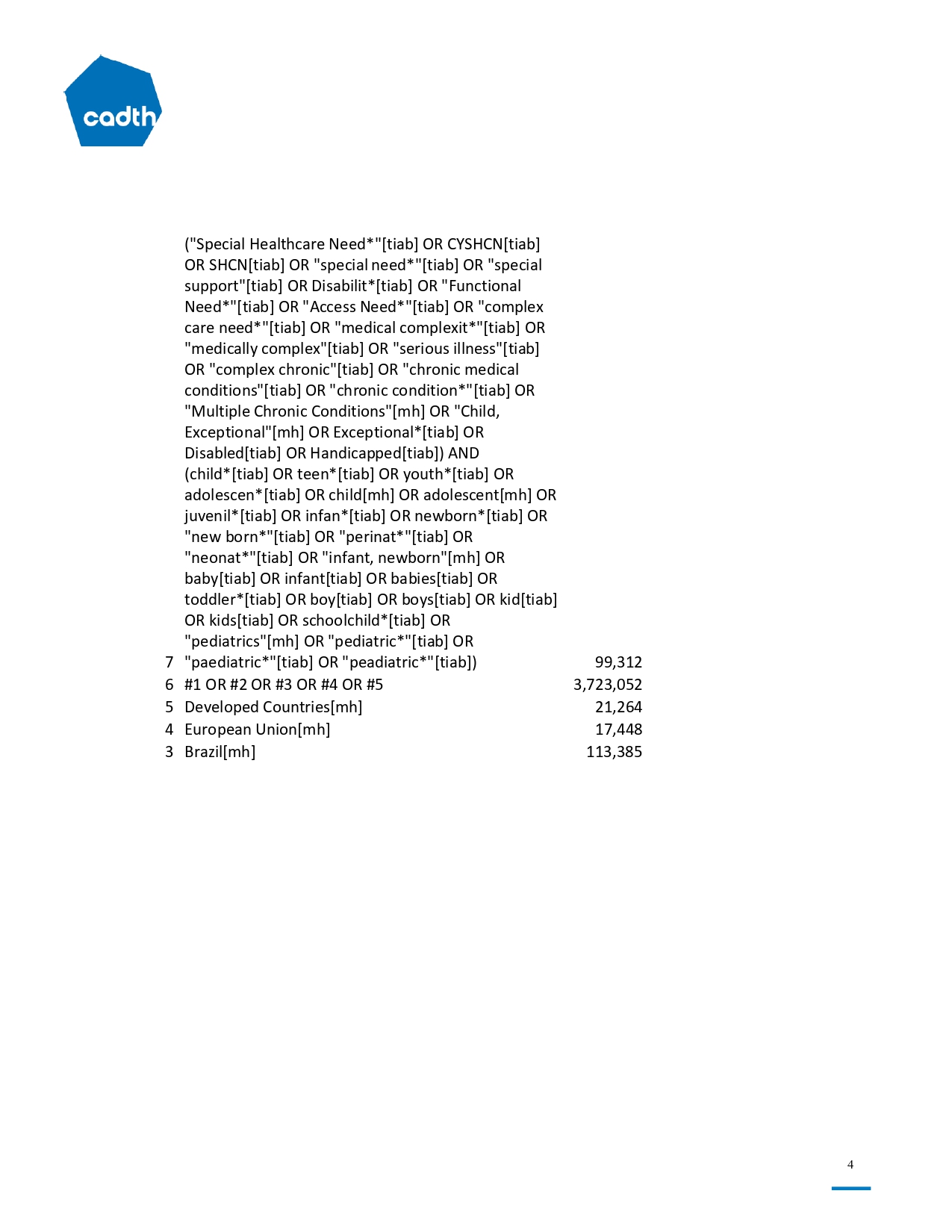

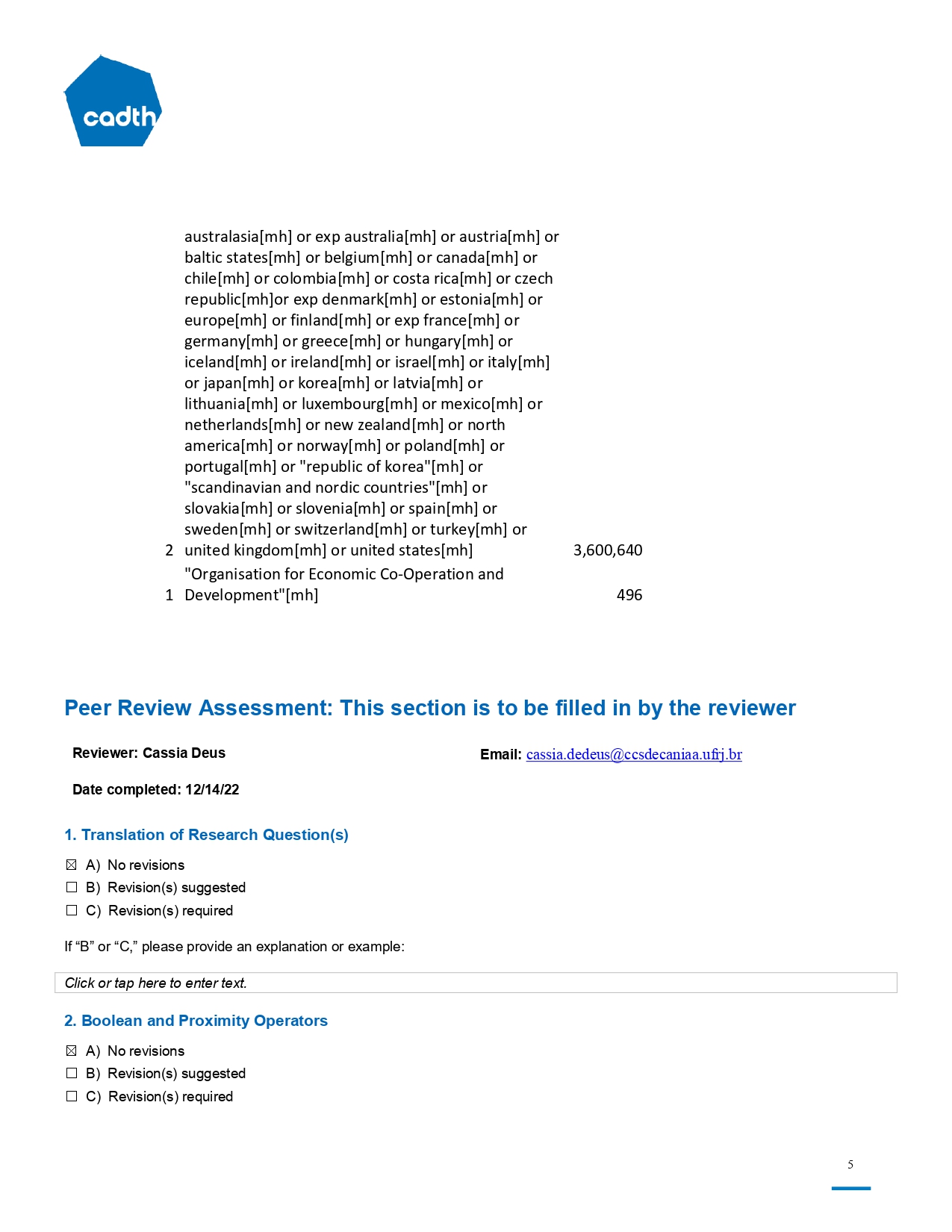

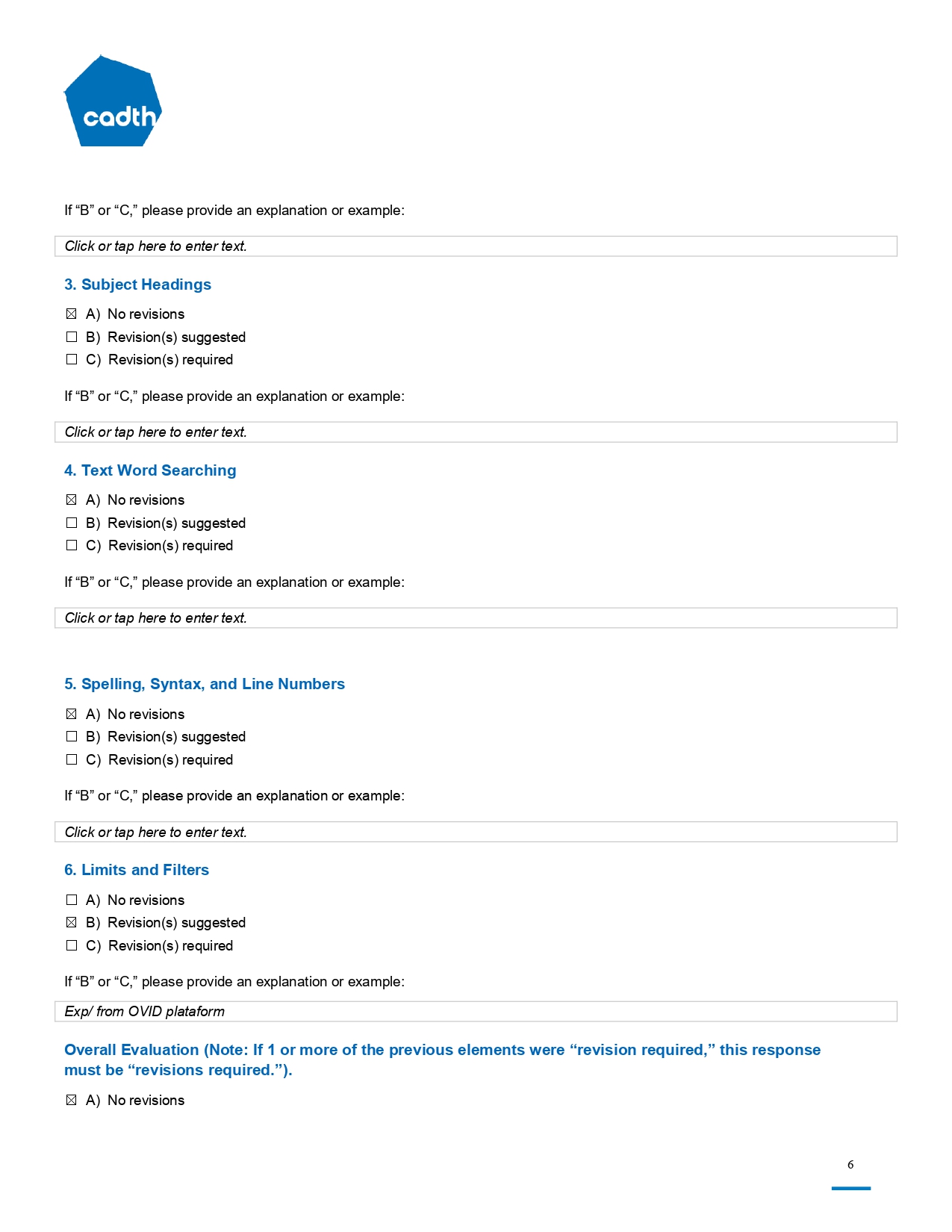

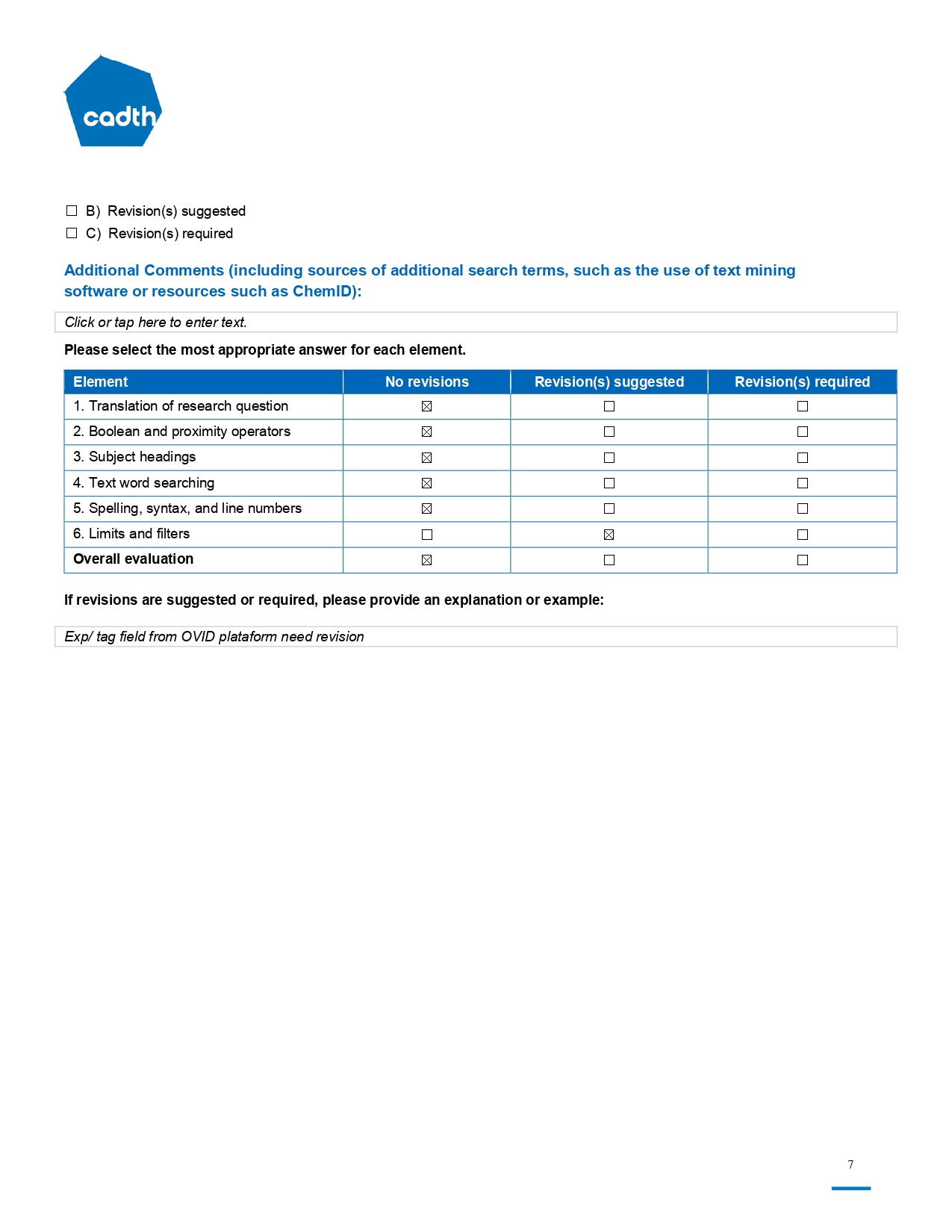

Supplement: S2 Fig — (DOCX) [file pone.0287939.s003.docx]
