# Supplementary material for: Public health policies for children and youth with special health care needs in OECD member countries and Brazil: A scoping review protocol
Source: PLoS One. 2023 Oct 11;18(10):e0287939. doi: 10.1371/journal.pone.0287939 (PMC10566681; doi:10.1371/journal.pone.0287939)
Supplement: Attachment — Submitted filename: Responses to Reviewers.docx [file pone.0287939.s004.docx]

Ana Larissa Gomes Machado, Ph.D.

Academic Editor

PLOS ONE

Dear Ms. Machado:

Thank you and the Reviewers for the favorable evaluation of our manuscript.

1) The manuscript has been revised to meet PLOS ONE style requirements and the three files being uploaded have been entitled “Response to Reviewers,” “Manuscript,” and “Revised Manuscript with Track Changes.”
2) The original title page has now been incorporated into the manuscript.
3) The ethics statement has been moved to the Methods section.
4) The list of references has been revised. No sources cited have been retracted.

The Reviewers’ remarks were addressed as follows:

Reviewer #1: no suggestions.

Reviewer# 2:

1) The principal objective has been rephrased.
2) Both the abstract and the main text now inform that DeCS (LILACS) is a multilingual thesaurus (the term employed on the DeCS website to describe itself).
3) Mention to the gray literature has been added to the abstract.
4) Articles not being available in full was removed as an exclusion criterion.
5) The controlled vocabularies used for each database and index have now been specified.
6) The suggestion not to mention the PCC Mnemonic in the Introduction and research question was accepted. This information has now been added to the Methods section.
7) Use of Rayyan software for identification and exclusion of duplicates has now been mentioned.

Sincerely,

The authors
